# A Streamlined Protocol for Molecular Testing of the *DMD* Gene within a Diagnostic Laboratory: A Combination of Array Comparative Genomic Hybridization and Bidirectional Sequence Analysis

**DOI:** 10.1155/2013/908317

**Published:** 2013-02-07

**Authors:** Renate Marquis-Nicholson, Daniel Lai, Chuan-Ching Lan, Jennifer M. Love, Donald R. Love

**Affiliations:** ^1^Diagnostic Genetics, LabPLUS, Auckland City Hospital, P.O. Box 110031, Auckland 1148, New Zealand; ^2^School of Biological Sciences, University of Auckland, Private Bag 92019, Auckland 1142, New Zealand

## Abstract

*Purpose*. The aim of this study was to develop a streamlined mutation screening protocol for the *DMD* gene in order to confirm a clinical diagnosis of Duchenne or Becker muscular dystrophy in affected males and to clarify the carrier status of female family members. *Methods*. Sequence analysis and array comparative genomic hybridization (aCGH) were used to identify mutations in the dystrophin *DMD* gene. We analysed genomic DNA from six individuals with a range of previously characterised mutations and from eight individuals who had not previously undergone any form of molecular analysis. *Results*. We successfully identified the known mutations in all six patients. A molecular diagnosis was also made in three of the four patients with a clinical diagnosis who had not undergone prior genetic screening, and testing for familial mutations was successfully completed for the remaining four patients. *Conclusion*. The mutation screening protocol described here meets best practice guidelines for molecular testing of the *DMD* gene in a diagnostic laboratory. The aCGH method is a superior alternative to more conventional assays such as multiplex ligation-dependent probe amplification (MLPA). The combination of aCGH and sequence analysis will detect mutations in 98% of patients with the Duchenne or Becker muscular dystrophy.

## 1. Introduction

The dystrophinopathies are a group of muscle disorders that are caused by mutations in the *DMD* gene [1]. The *DMD* gene encodes dystrophin, a glycoprotein that is present principally in muscle cells and forms part of the complex linking the cytoskeleton with the extracellular matrix [2]. Mutations that lead to a complete lack of dystrophin expression tend to cause the more severe Duchenne muscular dystrophy (DMD) phenotype, whereas mutations that lead to an abnormal quality or quantity of dystrophin result in the Becker muscular dystrophy (BMD) [3]. In addition, DMD-related X-linked dilated cardiomyopathy (DCM) occurs as a result of mutations that lead to a lack of functional dystrophin in cardiac muscles due to altered tissue-specific transcription or alternative splicing [4].

More than 5,000 mutations have been identified in individuals with DMD or BMD [5, 6]. These pathogenic mutations are highly variable and run the full gamut from deletion of the entire gene, to deletion or duplication of one or more exons, to small deletions or insertions, and to single-base pair changes. Deletions and duplications account for 60–70% and 5–10%, respectively, of all cases [3]. Sequence variants (point mutations and small indels) are responsible for a further 25–35% of cases [7]. There are two hotspots for recombination within the gene, one at the 5′ end (exons 2–20) and the other at the distal end (exons 44–53), leading to a cluster of deletions and duplications at these locations [8, 9]. As a general rule, mutations that alter the reading frame correlate with DMD, whereas those that preserve the reading frame are associated with BMD [6, 10].

Molecular testing is useful to confirm a clinical diagnosis in affected males who are suspected to have a dystrophinopathy based on clinical signs and an elevated serum creatine kinase (CK) level and obviates the need for a muscle biopsy. The identification of the causative mutation in an affected individual also informs genetic counselling for the family and allows carrier and prenatal testing to be performed as appropriate [11].

We report here the development of a streamlined mutation screening protocol for the *DMD* gene in order to confirm a clinical diagnosis of affected males and to clarify the carrier status of female family members. This protocol involves the use of array comparative hybridisation (aCGH) in a primary screen for deletions/duplications, followed by bidirectional sequence analysis if no copy number change is found.

## 2. Materials and Methods

### 2.1. Patient Samples

A group of six individuals with known changes within the *DMD* gene were selected for the validation of the aCGH and sequencing assays. This group included four male individuals with a confirmed diagnosis of DMD: two as a result of nonsense mutations in the *DMD* gene (sequence analysis performed at an overseas laboratory) and two as a result of multiexon deletions (detected in our laboratory by multiplex ligation-dependent probe amplification, MLPA). The fifth individual was the pregnant female carrier of a deletion in the *DMD* gene, who was hoping to have prenatal testing carried out at the appropriate point in her pregnancy. Her initial testing had also been performed using MLPA. The sixth was the deceased proband of a family with multiple female members who wanted to clarify the familial mutation and subsequently proceed to carrier testing. Following his death, twenty years ago, Southern blot analysis of the *DMD* gene with cDNA probes (performed at an overseas laboratory) had identified a heterozygous duplication involving exons 10 and 11 in DNA extracted from his sister, but this change had never been confirmed in the proband. The only source of DNA available for this individual was a Guthrie card collected at the time of routine newborn screening.

Following full validation of the aCGH and sequencing procedures, a further eight individuals were analysed. This analysis involved carrier testing of three female family members of the probands who were analysed as part of the validation group, prenatal testing of the male foetus of the female carrier mentioned above, and routine diagnostic testing of four males with a clinical diagnosis of dystrophinopathy.

The twelve peripheral blood EDTA samples and one chorionic villus sample (CVS) were referred to the Diagnostic Genetics section of LabPLUS, Auckland City Hospital, for diagnostic, carrier, or prenatal testing, as appropriate. A portion of the Guthrie card for the final individual in the validation group was provided by the National Testing Centre, which administers the collection and storage of these samples. Informed consent for genetic testing was given by each patient, or by the appropriate parent/guardian.

### 2.2. DNA Extraction

Genomic DNA (gDNA) was extracted from peripheral blood EDTA samples using the Gentra Puregene DNA Extraction kit (Qiagen) and from the Guthrie card using the QIAmp DNA Miniblood Kit (Qiagen). Standard phenol/chloroform extraction followed by ethanol precipitation was used to isolate gDNA from chorionic villus cells.

### 2.3. Primer Design

The dystrophin gene mRNA sequence of interest was identified through the public UCSC genome browser page at http://genome.ucsc.edu; RefSeq accession number NM_004006.2. We used the primer design program BatchPD to design primers flanking each of the coding exons (1–79) of the *DMD* gene, including 50 base pairs of the flanking intronic regions (see Table 1 for primer sequences). BatchPD was designed to automate and streamline the primer design process by using the appropriate RefSeq accession number to interface with a range of available online tools and provide a standardised summary output of the most relevant information (such as suitable primer sequences, the genomic coordinates of these primers, and the size of the amplicons produced) [12]. Included in the output is a list of primer sequences that are formatted for easy entry into SNPCheck, the online software tool available from the National Genetic Reference Laboratory, Manchester (https://ngrl.manchester.ac.uk/SNPCheckV3/snpcheck.htm), which can be used to check the primers for underlying single nucleotide polymorphisms. The primers were tailed with M13 sequences and were synthesised by Integrated DNA Technologies Inc.

Primer designs that failed PCR amplification or produced unsatisfactory amplification results were identified (see Table 1: highlighted primers) and required redesigns. The redesign process involved returning to the BatchPD results and identifying alternative primer designs. If the sequences of these designs differed enough from the original designs, then the sequences were subjected to SNPCheck and, passing that, the primers were ordered and assessed by PCR amplification. If the alternative primer sequences were too similar to the original primers, then a custom primer design workflow was undertaken. This workflow involved using Primer3 software (http://frodo.wi.mit.edu/primer3/) and the user interface to define the region to be amplified, followed by SNPCheck and, if the primers were found not overlie SNPs, then PCR evaluation. In the case of exon 68 that contains a repetitive sequence immediately 3′ of the splice donor site, a reverse sequencing primer was designed using Primer 3 software (see Table 1).

### 2.4. PCR

PCR was performed using 1 U Faststart Taq DNA polymerase (Invitrogen Ltd.), 50 ng genomic DNA, 2 mM MgCl_2_, 0.8 *μ*M forward and reverse primers, with the following cycle conditions: 95°C for 4 min, 35 cycles of 94°C for 45 s, 60°C for 30 s, 72°C for 30 s, and a final extension at 72°C for 10 min.

### 2.5. Sequencing

5 *μ*L of each PCR was cleaned with ExoSAP-IT (Affymetrix) prior to bidirectional DNA sequencing using M13 forward and reverse primers and BigDye Terminator v3.0 (Applied Biosystems Ltd.). 5 *μ*L of sequenced product was purified using the BigDye XTerminator Purification Kit (Applied Biosystems Ltd.). Purified product was then subjected to capillary electrophoresis using the Applied Biosystems model 3130xl Genetic Analyzer.

The analysis of sequence traces was performed using Variant Reporter v1.1 (Applied Biosystems). Genebank NM_004006.2 was used as the reference sequence, with cDNA number + 1 corresponding to the A of the translation initiation codon (codon 1). Variant Reporter uses advanced algorithms and quality metrics to automate the detection of variants and to streamline the analysis process. 

### 2.6. Dosage Analysis

A Roche NimbleGen 12x135K Custom CGH Array was used for dosage analysis. This bespoke CGH array has been designed to interrogate the coding regions of sixty-six genes of interest to our laboratory [13]. 

Two hundred and fifty nanograms of gDNA were processed according to the manufacturer's instructions (NimbleGen Array User's Guide: CGH and CNV Arrays v6.0; http://www.nimblegen.com). In brief, extracted gDNA from samples and Promega controls was denatured in the presence of a Cy3- (test-) or Cy5- (control-) labelled random primers and incubated with the Klenow fragment of DNA polymerase, together with dNTPs (5 mM of each dNTP), at 37°C for 2 hours. The reaction was terminated by the addition of 0.5 M EDTA (21.5 *μ*L), prior to isopropanol precipitation and ethanol washing. Following quantification, the test and sex-matched control samples were combined in equimolar amounts and applied to one of the twelve arrays on the microarray slide. Hybridisation was carried out in a Roche NimbleGen Hybridisation Chamber for a period of 48 hours. Slides were washed and scanned using a NimbleGen MS 200 Microarray Scanner. Array image files (.tif) produced by the MS 200 Data Collection Software were imported into DEVA v1.2.1 (Roche NimbleGen Inc.) for analysis. Each genomic region exhibiting a copy number change within the *DMD* gene was examined using the UCSC genome browser (http://genome.ucsc.edu) to determine the location and significance of the change. Data was filtered using a log_2_ ratio threshold of less than −0.4 over 6 probes for a deletion and greater than 0.4 over 15 probes for a duplication.

## 3. Results

### 3.1. Validation of Known Mutations

We developed a streamlined mutation-screening protocol for the *DMD* gene. This involved the use of a bespoke CGH array for the deletion/duplication analysis followed, if necessary, by sequence analysis for the detection of point mutations and small indels. As part of the sequencing pipeline, we used a new bioinformatic tool, BatchPD, for efficient primer design. BatchPD successfully designed primers to amplify all coding exons of the *DMD* gene under the same PCR conditions and the quality of the double-stranded sequence produced was consistently high.

**Figure 1:**
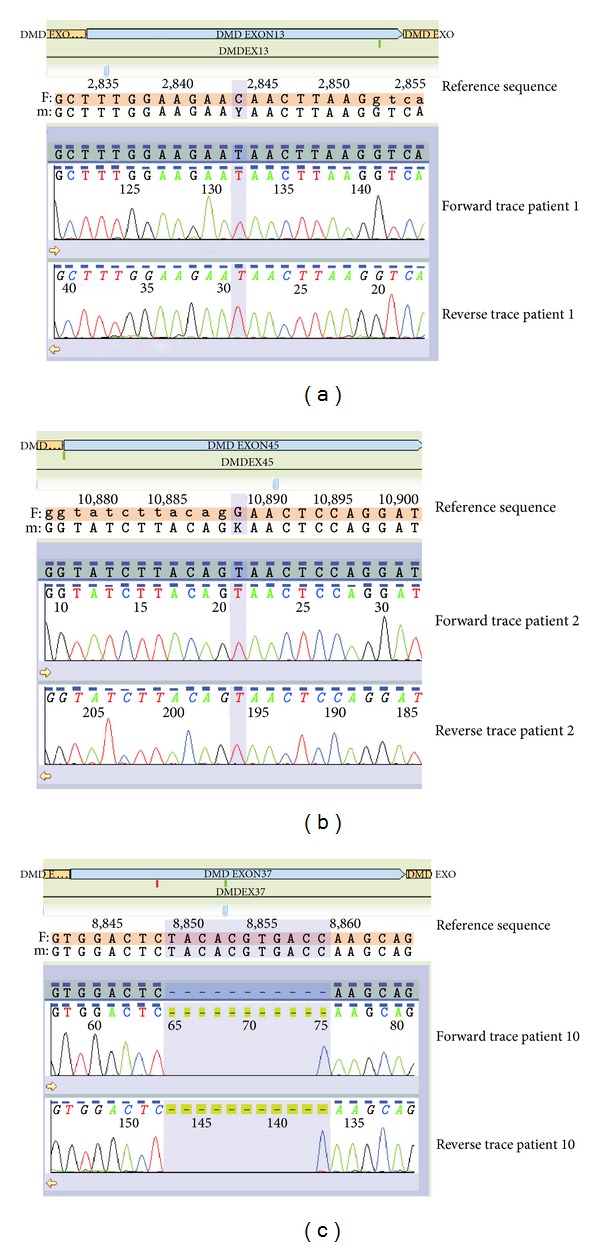
Sequence electropherograms. ((a), (b), and (c)) The c.1594C>T (p.Gln532X), c.6439G>T (p.Glu2147X), and c.5199_5209del (p.Thr1734SerfsX10) *DMD* gene mutations identified in patients 1, 2, and 10, respectively.

In order to validate the screening protocol, we analysed six patients with known mutations in the *DMD* gene. We were able to accurately identify the known changes in all six patients (patients 1–6, Table 2 and Figure 1), including detection of the familial duplication in the individual for whom the only remaining source of DNA was an archived Guthrie card. 

### 3.2. Additional Analysis

Four males with a clinical diagnosis of dystrophinopathy were referred for routine diagnostic testing. Patient 8 had previously undergone multiplex PCR [3] for deletions several years earlier and gDNA had more recently been sent away for full sequence analysis; both of these assays were normal. Array CGH revealed a duplication of exon 12 of the *DMD* gene. Further analysis (using the Reading-frame Checker, available online at http://www.dmd.nl/) predicted that this duplication would be in frame, which is consistent with the observed BMD phenotype. Array CGH also identified a mutation in two of the remaining three patients with a clinical diagnosis of the Duchenne muscular dystrophy. An out-of-frame deletion of exons 45–48 (inclusive) was detected in patient 9. A deletion of six probes was identified in patient 10. This intraexonic deletion was at the lower limit of the size threshold for analysis and involved a small portion of exon 37 only. Sequence analysis of exon 37 confirmed a hemizygous deletion of 11 base pairs within the exon, c.5199_5209del (p.Thr1734SerfsX10) (Figure 1). Although this mutation has not, to the best of our knowledge, been reported in the literature or in mutation databases, it results in premature termination of translation and truncation of the protein and is therefore consistent with the clinical diagnosis of DMD. No mutations were identified using the combination of dosage and sequence analysis for the remaining patient (patient 11).

DNA extracted from a CVS from patient 5 was received at 11 weeks' gestation. A rapid aneuscreen indicated that the foetus (patient 7) was male, so prenatal BMD testing was requested. Following exclusion of maternal cell contamination, aCGH was performed. The familial deletion of exons 45–47 (inclusive) was identified in the foetus and the decision was made to terminate the pregnancy. 

The final three patients analysed were referred for carrier testing. The familial deletion was identified in two of the three. The duplication previously identified in the affected son of the third individual was not detected on screening of her gDNA, but germline mosaicism could not be excluded. 

## 4. Discussion

The current European Molecular Genetics Quality Network (EMQN) Best Practice Guidelines on molecular diagnostics in the Duchenne/Becker muscular dystrophies recommend an initial screen for deletions and duplications, followed by a screen for point mutations if the clinical diagnosis is certain but a deletion/duplication has not been found [11]. It is acknowledged by the EMQN group that the approach used in different centres may vary depending on the availability of tests and facilities, as well as economic factors [11]. The *DMD* gene is the largest human gene yet described, comprised of 79 coding exons and 8 tissue-specific promoters distributed across approximately 2.2 Mb of genomic sequence [7]. Given the large size of the gene, it has historically been challenging for both technical and financial reasons to perform full sequence analysis [14]. As a consequence, numerous methods have been used to scan the *DMD* gene for point mutations and small indels, including single-strand conformation polymorphism [15], denaturing high performance liquid chromatography [14], the protein truncation test [16], and high-resolution melting curve analysis [17]. Although these mutation scanning methods are a low-cost alternative to sequencing and, therefore, may be more accessible to a small laboratory, the cost of sequencing has reduced appreciably over the last few years and it is now more efficient and cost-effective to perform full sequence analysis as the principle technique.

The process of designing suitable primers and optimising the PCR conditions for a sequencing assay can be very labour intensive, and the effort required increases as the size of the gene and number of exons increases. We employed a new primer design programme, BatchPD, to design primers which would allow us to amplify all 79 coding exons of the *DMD* gene under a single set of PCR conditions. As part of the validation process, the developers of the BatchPD programme designed primers for all 9000 human RefSeq genes, and these primers (as well as the source code for the programme) are freely available online [12]. We found that the use of BatchPD radically reduced the amount of time required to design the primers, and the optimisation process was simple, merely requiring a trial of the primers with and without GC-Rich solution (Roche Diagnostics Ltd.). In addition, as Next Generation Sequencing platforms become more affordable and accessible, it will be possible to retrofit these M13-tailed primers with user-defined custom tails, allowing an easy transition from capillary-based to next generation techniques.

Array CGH has recently been recognised as a superior method to the other most widely used dosage technique, MLPA [13, 18–22]. Compared to MLPA, aCGH allows the interrogation of intronic as well as exonic regions; hence, breakpoints can be mapped more accurately [11]. It can also be used to characterise some inversions and complex rearrangements, thereby offering a slightly higher mutation detection rate compared to MLPA and other purely exon-focused dosage assays [18, 19]. The aCGH process eliminates the risk of false positives that can occur as a result of polymorphisms under primer binding sites [11]. This risk is inherent in all PCR-based techniques and necessitates confirmation of single exon changes by a second technique or using a different set of primers [11]. The array design we report here is particularly cost effective, since twelve patients can be tested simultaneously for copy number changes in a range of genes, allowing efficient batching of patient samples. The overlapping probes tiling the exons in this array also mean that exceptionally high resolution can be achieved. Using this bespoke CGH array, we were able to confidently detect the full spectrum of dosage changes responsible for DMD and BMD: deletions and duplications involving multiple exons, single exon changes, and even an intraexonic deletion of only 11 base pairs.

## 5. Conclusions

In summary, the *DMD* gene testing protocol we report here is one that meets current best practice guidelines and can be implemented in a small diagnostic laboratory. Each of the techniques is robust and cost effective and allows for a comprehensive analysis of gDNA extracted from a range of sample types, including peripheral blood, chorionic villus tissue, and dried blood spots. The pipeline of dosage analysis using aCGH followed by full sequence analysis will detect mutations in approximately 98% of patients with the Duchenne or the Becker muscular dystrophy [11]. For the remaining 2% of patients, it is likely that a more complex rearrangement or deep intronic variant is involved, and either an RNA-based method or whole genome sequencing will be required.

## Figures and Tables

**Table 1:** Primers designed to amplify the coding exons of the *DMD* gene (NM_004006.2).

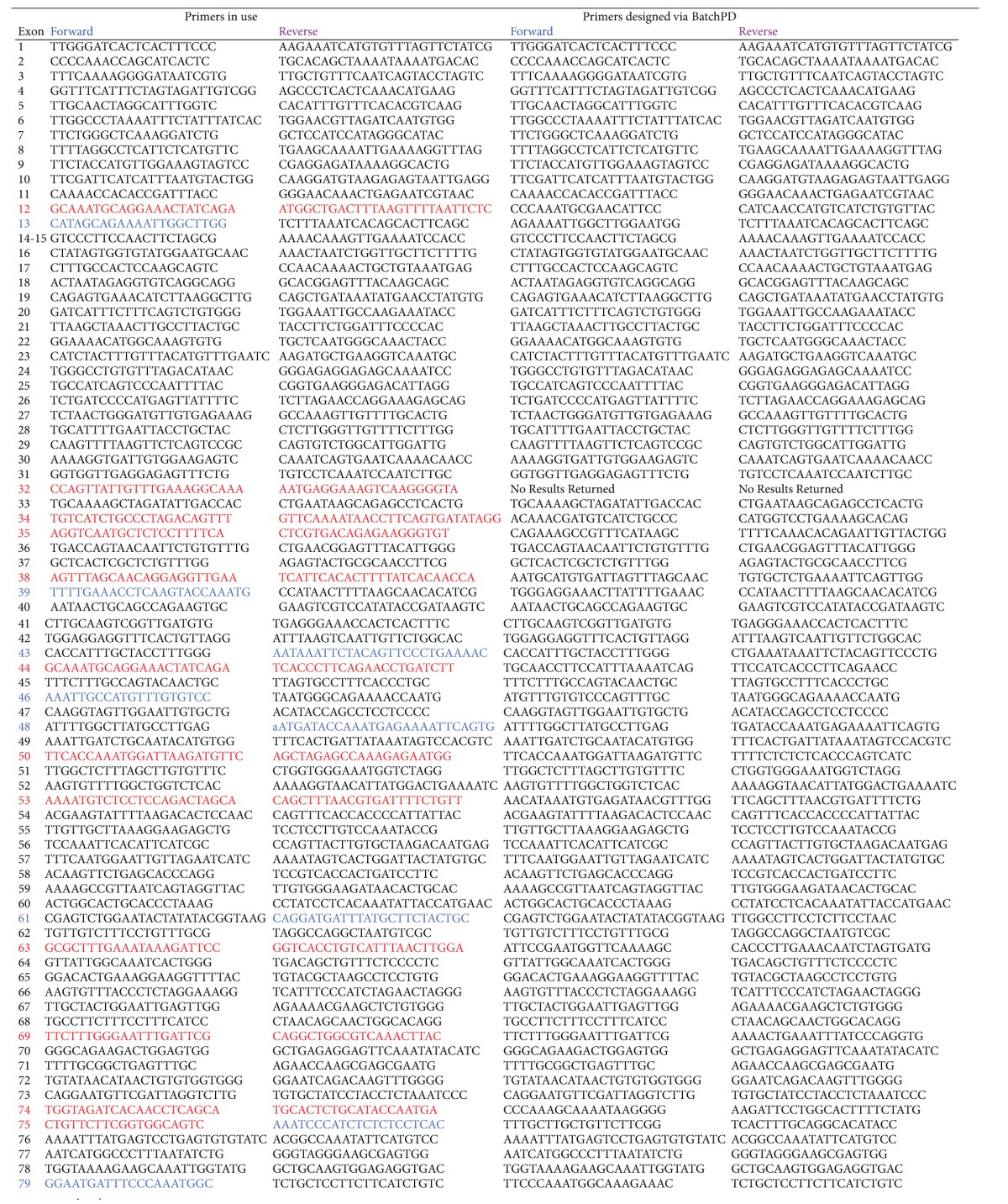

Internal reverse sequencing primer: 68_R1 ggttcctaatacctgaatccaatg.

Key: BatchPD alternative design (shown in blue); Custom primer re-design (shown in red).

**Table 2:** Mutations within the *DMD* gene—all patient samples.

Patient	Referral reason/phenotype	Genotype	Previous testing method
1	Duchenne muscular dystrophy	c.1594C>T (p.Gln532X)	Sanger sequencing
2	Duchenne muscular dystrophy	c.6439G>T (p.Glu2147X)	Sanger sequencing
3	Duchenne muscular dystrophy	Deletion exons 3–7	MLPA
4	Duchenne muscular dystrophy	Deletion exons 3–44	MLPA
5	Carrier of Becker muscular dystrophy	Deletion exons 45–47	MLPA
6	Duchenne muscular dystrophy	Duplication exons 10, 11	Southern blot
7	Prenatal test for Becker muscular dystrophy (foetus of patient 5)	Deletion exons 45–47	
8	Becker muscular dystrophy	Duplication exon 12	
9	Duchenne muscular dystrophy	Deletion exons 45–48	
10	Duchenne muscular dystrophy	c.5199_5209del (p.Thr1734SerfsX10)	No previous testing performed
11	Becker muscular dystrophy	No mutation detected	
12	Carrier of Duchenne muscular dystrophy (mother of patient 3)	Heterozygous deletion exons 3–7	
13	Carrier of Duchenne muscular dystrophy (mother patient 4)	Heterozygous deletion exons 45–47	
14	Carrier of Becker muscular dystrophy (mother of patient 8)	Duplication exon 12 NOT detected	
